# Selective vs stepwise removal of deep carious lesions in primary molars: 24 months follow-up from a randomized controlled trial

**DOI:** 10.1007/s00784-020-03536-6

**Published:** 2020-08-28

**Authors:** Karim Elhennawy, Christian Finke, Sebastian Paris, Seif Reda, Paul-Georg Jost-Brinkmann, Falk Schwendicke

**Affiliations:** 1 Department of Orthodontics, Dentofacial Orthopedics and Pedodontics, Charité – Universitätsmedizin Berlin, corporate member of Freie Universität Berlin, Humboldt-Universität, and Berlin Institute of Health, Aßmannshauser Str. 4-6, 14197 Berlin, Germany; 2 Department of Operative and Preventive Dentistry, Charité – Universitätsmedizin Berlin, corporate member of Freie Universität Berlin, Humboldt-Universität, and Berlin Institute of Health, Aßmannshauser Str. 4-6, 14197 Berlin, Germany; 3 Department of Oral Diagnostics, Digital Health and Health Services Research, Charité – Universitätsmedizin Berlin, corporate member of Freie Universität Berlin, Humboldt-Universität, and Berlin Institute of Health, Aßmannshauser Str. 4-6, 14197 Berlin, Germany

**Keywords:** Caries, Costs, Dentin, Partial removal, Restoration, Two-step removal

## Abstract

**Objectives:**

For well-defined deep (> 2/3 dentin extension) carious lesions, selective (SE) or stepwise (SW) carious tissue removals have been recommended, while there is limited comparative evidence for both. We compared SE and SW over 24 months in a randomized controlled trial.

**Methods:**

A two-arm superiority trial was conducted comparing SW/SE in primary molars without pulpal symptoms but well-defined deep lesions. Seventy-four children (1 molar/child) aged 3–9 years were recruited. In a first step, peripheral carious tissue was removed until hard dentin remained, while in proximity to the pulp, leathery dentin was left. An adhesive compomer restoration was placed and restorations re-examined after 6 months. In SW, re-entry and removal to firm dentin was conducted pulpo-proximally, followed by re-restoration. Molars were re-evaluated for a total of 24 months. Our primary outcome was success (absence of restorative/endodontic complications or pulp exposures). Secondary outcomes included total treatment and opportunity costs and restoration quality, assessed using modified USPHS criteria.

**Results:**

After 24 months, 63 molars (31 SE, 32 SW) were re-assessed. Four failures occurred (2 exposures in SW; 2 pulpal complications in SE, 1 of them leading to extraction, *p* > 0.05). Restoration integrity was satisfying in both groups (USPHS A/B/C in 21/8/0 SE and 23/7/0 SW, *p* > 0.05). Treatment and opportunity costs were significantly higher in SW than SE (mean 171 ± 51 vs. 106 ± 90; *p* < 0.001).

**Conclusions:**

After 2 years, SE and SW showed similar efficacy for managing deep carious lesions in primary molars. The higher costs for SW should be considered during decision-making.

**Clinical significance:**

In primary molars with well-defined deep carious lesions SE was less costly and similarly efficacious like SW. From a cost and applicability perspective, SW may need to be indicated restrictively, e.g., for very deep (> 3/4 dentin extension) lesions only.

**Trial registration:**

ClinicalTrials.gov Identifier: NCT02232828

## Introduction

When managing deep carious lesions, especially in primary teeth, the risk of pulp exposure and complications is high, and managing these complications usually involves endodontic, surgical, or orthodontic follow-up treatments [1–3]. To avoid pulp exposure and complications, selective or stepwise carious tissue removal (SE, SW), where carious dentin is sealed beneath a restoration permanently or in-between treatment steps, has been recommended over conventional, non-selective removal, which aims to remove all carious dentin. However, there is limited data comparing SE and SW. When initiating the present study, only one three-arm study involving 63 primary teeth had compared both therapies for managing deep carious lesions, i.e., those involving the inner third of the dentin [4]. This study found both strategies to show similar risks of pulp exposures and complications, but did not at all assess restorative complications or compared the costs of both treatments, which will be very different initially given the second step being required for SW, but may be similarly long term in case SE shows more restorative complications than SW, for example, [5, 6].

Hence, a randomized trial was performed, comparing the success and survival, the initial and follow-up treatment costs, and the quality of restorations placed after SE versus SW in well-defined deep carious lesions in primary molars. Our primary hypothesis was that the success differs significantly between SE and SW. In a 1-year interim analysis [7], we showed that success, survival, patients’, parents’, and dentists’ subjective evaluation did not significantly differ. Notably, both the initial and long-term cost were significantly higher in SW than SE. In the present analysis of this trial after 24 months, we report on success, survival and costs, as well as the restoration quality according to modified USPHS criteria.

## Methods

### Study design

**Fig. 1:**
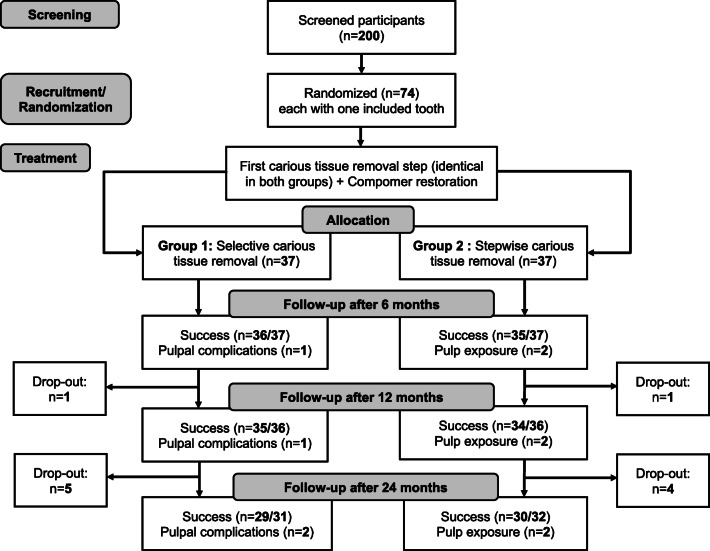
Flowchart of the study

The 1-year interim results from this study have been reported before [7]; this is the second interim analysis before the final results will be published after 36 months. This study is a two-arm, parallel-group, single-blinded, randomized controlled superiority trial, conducted at the dental clinic of Charité - Universitätsmedizin Berlin, Germany. The study flow is summarized in Fig. 1. The study has been approved by the ethics committee of the Charité - Universitätsmedizin Berlin (EA4/057/14) and registered at ClinicalTrials.gov (NCT02232828). The study was originally planned as multi-center study, the respective protocol has been published [8]. Deviations from the protocol have been described elsewhere [7], but will be mentioned below.

### Setting and participants

Recruitment was conducted from routine examinations of in-house and referred patients. We included children aged 3–9 with minimum one primary molar with a deep but well-defined carious lesion. The molar was supposed to be vital, determined via thermal (cold) sensitivity testing, vital, clinically and radiographically non-symptomatic, and retainable. The carious lesion was required to radiographically extend into the inner third of the dentin (D3) and show signs of activity [9]. We included lesions involving both only the occlusal or the occlusal and one proximal (mesial or distal) surface (i.e., one- or two-surfaced lesions). Parental consent was required and patients’ cooperation for treatment under no or local anesthesia was to be expected. Patients with systemic diseases or disabilities, known allergies to the used materials, and teeth expected to exfoliate within the next 18 months were excluded.

### Sample size

Sample size calculation for this trial was conducted for our primary outcome parameter, success (absence of endodontic or restorative complications). Based on a previous study on permanent teeth, we expected a hazard ratio of 1.3 [10] of SW versus SE, with *α* = 0.05 and 1-*β* = 0.9. Factoring in drop-out and considering possible subgroup analyses being required, we had aimed to include 192 patients in multiple centers. As eventually, only one center participated, we could not realize this sample size and terminated recruitment after 15 months, recruiting 74 patients. We hence have to assume that our trial might be under-powered. Note, however, that in the present analysis, events (complications) were balanced between groups, and it is highly unlikely that statistical power will have at all affected our conclusions.

### Interventions

A full intraoral examination, caries risk assessment [11] and dental anxiety measurement [12], was performed in the first visit (T0). Treatment was provided in the second visit (T1). In case that more than one primary molar met the inclusion criteria, the tooth to be included to the study was chosen based on random number tables prior to conducting the treatment (the other teeth requiring treatment were managed within routine care). Removal of enamel and cavity preparation was performed using water-cooled diamond instruments. Carious tissue removal in the periphery was performed using low-speed rose head burs until hard, dry dentin remained. In proximity to the pulp, carious tissue was removed until leathery, slightly moist dentin remained. The operators were calibrated as to this endpoint prior to the study on extracted teeth. Moisture control was performed using cotton rolls. After excavation, a self-etch adhesive (G-aenial bond, GC, Bad Homburg, Germany) was rubbed in for 10 s, gently dried for 5 s and light-cured using an LED curing light (Satelec as part of Sirona Teneo, Dentsply) with a maximum light output of 1190 mW/cm^2^ for 20 s according the manufacturer’s instructions. Then, a polyacrylic acid-modified composite (compomer) material (Dyract, Dentsply, Konstanz, Germany) was placed in 2-mm increments, which were light-cured for 20 s as described. For lesions extending proximally, a Tofflemire matrix (Henry Schein Dental, Langen, Germany) was employed, since it is the conventially used matrix in the dental school. Note that this may come with some disadvantages regarding the construction of a bulky proximal surface, while its applicability was found superior over segment matrices, which are not regularly accepted given the separator ring being uncomfortable for children without anesthesia. The restoration was finished with a fine grid round diamond stone (Komet Dental, Lemgo, Germany) and polished with a restoration polishing system (Enhance PoGo, Dentsply).

The first treatment visit was performed identically in both groups, by one of two calibrated operators, as described. After 6 months (T2), the follow-up examination was performed blinded for groups, and only afterwards the allocation was revealed to the operator. If allocated to SW, the restoration was removed and carious dentin excavated as described until only firm dentin remained in proximity to the pulp. A new restoration was then provided adhesively as described. In SE, no further treatment was conducted.

### Data collection and follow-up visits

Data collection has been described in detail before [7]. Briefly, at T1, the subjective assessment of the treatment by the patients was recorded immediately after the excavation using a visual analog scale (VAS) (score 0–10). The subjective assessment of the parent (grade 1–6) and the dentist (grade 1–6) was also measured. We do not report on these aspects again. To later on determine the direct and indirect costs of both treatments, we recorded traveling and waiting times as well as the time needed for treatment. We also recorded the staff who provided the therapy and the material used. At T2 (6 months after the initial treatment), T3 (12 months), T4 (18 months), and T5 (24 months), we further recorded if the tooth had been re-treated elsewhere (yes/no), exfoliated (yes/no), or showed any sensitivity and symptoms. Moreover, the restoration integrity was measured by one examiner who had been calibrated prior to the study using modified USPHS criteria, as shown in Table 1 [13]. The examiner was independent from the operators and blinded for the group allocation.

**Table 1:** Modified united states public health service (USPHS) Ryge criteria for direct clinical evaluation of restoration

Category	Scores	Scoring method	Criteria
Color match	Alpha (A)	Visual inspection	The restoration appears to match the shade and translucency of adjacent tooth tissues.
	Bravo (B)	Visual inspection	The restoration does not match the shade and translucency of adjacent tooth tissues, but the mismatch is within the normal range of tooth shades.
	Charlie (C)	Visual inspection	The restoration does not match the shade and translucency of the adjacent tooth structure, and the mismatch is outside the normal range of tooth shades and translucency.
Marginal discoloration	Alpha (A)	Visual inspection	There is no visual evidence of marginal discoloration.
	Bravo (B)	Visual inspection	There is visual evidence of marginal discoloration at the junction of the tooth structure and the restoration, but the discoloration has not penetrated along the restoration in a pulpal direction.
	Charlie (C)	Visual inspection	There is visual evidence of marginal discoloration at the junction of the tooth structure and the restoration that has penetrated along the restoration in a pulpal direction.
Marginal integrity	Alpha (A)	Visual inspection and explorer	The explorer does not catch when drawn across the surface of the restoration.
	Bravo (B)	Visual inspection and explorer	The explorer catches and there is visible evidence of a crevice, which the explorer penetrates.
	Charlie (C)	Visual inspection and explorer	The explorer penetrates crevice defect extended to the dento-enamel junction.
Anatomic contour	Alpha (A)	Visual inspection and explorer	The restoration is a continuation of existing anatomic form or is slightly flattened.
	Bravo (B)	Visual inspection and explorer	A surface concavity is evident.
	Charlie (C)	Visual inspection and explorer	There is a loss of restorative substance such that a surface concavity is evident and the base and/or dentin is exposed.
Surface texture	Alpha (A)	Explorer	Surface texture similar to polished enamel as determined by means of a sharp explorer.
	Bravo (B)	Explorer	Surface texture gritty or similar to a surface subjects to a white stone or similar to a composite containing supramicron-sized particles.
	Charlie (C)	Explorer	Surface pitting is sufficiently coarse to inhibit the continuous movement of an explorer across the surface.

In case of pulpal exposures, vital amputation (pulpotomy) was performed, with the pulp chamber ceiling being removed, hemostasis using sterile cotton pellets and saline for 4–5 min followed by ferric-sulfate 15.5% (Astringedent, Ultradent, Köln, Germany) for 10–15 s, and placement of a calcium hydroxide cement (Dycal, Dentsply). Molars with amputated pulps were restored using preformed stainless steel crowns (3M, Neuss, Germany). They hence do not appear in the restoration performance analysis using USPHS criteria. In case pulpotomy was not indicated, molars were extracted and a customized fixed space maintainer placed. Time, staff, and materials used as well as travel and waiting times were also recorded for re-treatments to capture the sequels of both SE and SW at T3–5.

### Allocation and blinding

Sequence generation was performed using a simple random number table (no block randomization). Allocation concealment was performed via sealed opaque envelopes; deconcealment was performed at T2 (i.e., after 6 months) as described. Clinical follow-up examinations were performed by a dentist blinded to the allocation. Operator blinding during the second removal step as well as blinding of patients was not possible, but patients were informed not to reveal treatment allocation to the examiner during follow-up examinations.

### Outcomes and outcome measures

The primary outcome was success, i.e., the absence of endodontic or restorative complications. Secondary outcomes included (1) survival (i.e., not requiring extraction); (2) subjective assessment by patients, dentists, and parents; (3) restoration integrity as per modified USPHS criteria [13]; and (4) treatment and opportunity costs. For the latter, and in brief, a societal perspective was used, with direct medical and non-medical costs and indirect (opportunity) costs being considered. The horizon of our analysis was 24 months; to account for time preference and the opportunity cost of capital, a 3% annual discount was applied [14]. For cost estimation, the unit costs and the number of units consumed were employed. Costs for staff, the dental office (including rent, electricity, heating, and unit deprecation) were estimated based on hourly mean gross dental practice costs in Germany [15]. Material unit costs as well as laboratory costs were estimated based on market prices in 2015/16. Opportunity costs were calculated by applying a mean gross hourly wage in Berlin in 2017, including social insurance contributions [16], to both traveling and waiting costs for initial and re-treatments. Costs estimation and reporting followed the CHEERS guidelines [17].

### Statistical analysis

Statistical evaluation was performed using SPSS 20.0. Two-sided independent *t* tests or Mann-Whitney *U*-tests and chi-square tests were used for pairwise comparisons. Level of significance was set at *p* < 0.05.

## Results

The study flow is shown in Fig. 1. We recruited a total of 74 children (36 girls, 38 boys), each with one molar requiring treatment. Children’s mean (SD) age was 6.3 (1.5) years. No significant differences in baseline characteristics between groups were detected (Table 2).

**Table 2:** Characteristics of the participants. SE selective, SW stepwise carious tissue removal. No significant differences were observed (*p* > 0.05)

Item	SE	SW
*n*	37	37
Age in years (mean, SD)	6.3 (1.5)	6.3 (1.9)
Gender (male/female)	16/21	23/14
Cooperation (mean, SD)	3.0 (0.8)	3.3 (0.8)
Caries risk (low/middle/high)	2/12/23	2/13/22
Dental arch (upper/lower)	17/20	15/22
Primary molar (1^st^/2^nd^)	12/25	14/23
Surfaces (1/2)	20/17	17/20

During initial therapy, pulp exposure occurred in two SW molars, both at the second step. Both molars received pulpotomy and stainless steel crowns. No exposures occurred in SE. During follow-up, six SE and five SW molars were lost, respectively. Of the remaining 31 SE molars, two experienced endodontic complications during follow-up; one leading to extraction and placement of a space holder, the other to pulpotomy and placement of a stainless crown. No complications occurred in SW. Both initial and follow-up treatment and opportunity as well as overall costs were significantly higher in SW than SE (*p* < 0.001, Table 3).

**Table 3:** Results of the trial. SE selective, SW stepwise carious tissue removal. Significant differences between groups (*p* < 0.05) are indicated in italics

Item	SE	SW
Pulp exposures	0/37	2/37
Lost teeth	1/31	0/32
Total complications after 24 months	2/31	2/32
Initial costs (Euro, mean (SD))	*68.4 (20.1)*	*132.9 (18.3)*
Total treatment costs after 24 months (Euro, mean (SD))	*85 (74)*	*141 (44)*
Total opportunity costs after 24 months (Euro, mean (SD))	*20 (20)*	*35 (11)*
Total costs after 24 months (Euro, mean (SD))	*106 (90)*	*176 (51)*

We further assessed the performance of restorations placed after SE and SW, respectively (Table 4). In SE, restorations showed a moderate deterioration at 18 and, more so, 24 months, according to USPHS scoring. At 24 months, total of 8/29 SE restorations scored “Beta,” the rest “Alpha.” Deteriorations occurred largely in three domains: margin discoloration, marginal integrity, and anatomic form. In SW, similar deteriorations were observed, mainly after 24 months, with a total of 7/30 SW restorations showing scoring “Beta,” the rest “Alpha” after 24 months (*p* > 0.05), again pertaining to margin discoloration, marginal integrity, and anatomic form. None of the groups showed any inacceptable restorations.

**Table 4:** Clinical scores of the restorations in both groups according to the modified Ryge criteria. No significant differences between groups were detected (*p* > 0.05)

		Baseline			6 months			12 months			18 months			24 months			Total restorations evaluated
	Score	A	B	C	A	B	C	A	B	C	A	B	C	A	B	C	
SE	Color match	29	0	0	29	0	0	29	0	0	29	0	0	29	0	0	29
	Marginal discoloration	29	0	0	29	0	0	29	0	0	25	4	0	24	5	0	29
	Marginal integrity	29	0	0	29	0	0	29	0	0	26	3	0	24	5	0	29
	Anatomic form	29	0	0	29	0	0	29	0	0	26	3	0	26	3	0	29
	Surface texture	29	0	0	29	0	0	29	0	0	29	0	0	29	0	0	29
	Total deteriorated restorations	0			0			0			7			8			
SW	Color match	30	0	0	30	0	0	30	0	0	30	0	0	30	0	0	30
	Marginal discoloration	30	0	0	30	0	0	30	0	0	28	2	0	24	6	0	30
	Marginal integrity	30	0	0	30	0	0	30	0	0	27	3	0	26	4	0	30
	Anatomic form	30	0	0	30	0	0	30	0	0	29	1	0	27	3	0	30
	Surface texture	30	0	0	30	0	0	30	0	0	30	0	0	30	0	0	30
	Total deteriorated restorations	0			0			0			5			7			

## Discussion

When managing deep carious lesions in primary molars, dentists conventionally relied on non-selective (complete) carious tissue removal, which was found to be associated with high risks of pulp exposure and complications like pulpitis [18]. Exposure and pulpitis can often be successfully addressed by pulpotomy, which is an efficacious, but also challenging and costly therapy in primary molars, as confirmed by a range of studies [19, 20]. Also in our study, pulpotomy was rather costly, mainly as the treatment itself, but also the subsequent placement of a stainless steel crown is more expensive than the initial placement of a direct restoration. To avoid pulpal exposures and complications, SW and SE have been recommended and evaluated against non-selective removal by a range of studies [21]. Both SE and SW come with a number of advantages and disadvantages: While SW may reduce the long-term risk of restorative complications by removing all carious tissue in the second step and hence improving restoration adhesion and stability, at least in theory, it requires a second treatment step, burdening children and generating treatment and opportunity costs [22, 23]. Moreover, SW has been found to suffer from high risks of failure in the period between steps if a temporary restoration material is employed, mainly as such material is often partially or totally lost if patients extend this period, leading to pulpal complications [24]. In our study, this risk was mitigated as we did not place a temporary restorative material for the time period bridging the treatment steps, but polyacrylic acid–modified composite. SE, in contrasts, does not require a second step and shows nearly zero risk of pulp exposure, but might come with higher risks of restorative complications, especially when larger amounts of carious tissue are sealed beneath the restoration [6, 25]. There is, as outlined, very limited data comparing both therapies against each other. In this trial, we tested if SW was superior over SE in managing well-defined deep carious lesions in primary molars. The assumption of superiority was justified given the additional efforts associated with SW. We further compared their initial and long-term treatment and opportunity costs. The present publication additionally reports on the restorative performance after SE and SW.

After 24 months of follow-up, we did not find SW and SE to differ significantly with regards to their success, we hence reject our primary hypothesis. This is in line with data comparing SW and SW in permanent teeth; there, SW led to significantly more pulp exposures, mainly in the second removal step [22], while exposures in SE were rare events [22]. In our study and during follow-up, SE was associated with two pulpal complications, one leading to extraction and the other to pulpotomy. No follow-up events were noted during SW. It will be relevant to see if, over the next year until the 36 months final follow-up, this trend continues. Based on the present data, the overall risk of failure was nearly identical in SW and SE.

In the present study, and for the first time, we compared the performance of restorations placed after SW and SE. We did not find significant differences after 24 months, and no restoration failed. Notably, though, restorations in SE deteriorated slightly earlier than those in SW, mainly in the domains of margin discoloration and integrity as well as anatomic form. It is conceivable that the residual carious lesion sealed beneath the restoration detrimentally affects restoration longevity by reducing the adhesive surface and the support against masticatory forces, resulting in margin stress and loss of integrity. It should, however, be born in mind that the restorations in SE were simply 6 months older than those in SW, which had been exchanged during the second treatment step. SW restorations showed similar deteriorations, but with concomitant delay of 6 months.

SW is associated with high additional efforts and costs both for the dental treatment but also for transport and time spent. Our study reflected on these costs during the initial but also follow-up treatments. SW showed significantly higher costs, mainly due to the second treatment step being required. The pulp exposures in SW further added to these costs, while the pulpal complications in SE also generated relatively high costs (for extraction and space holder, or pulpotomy and stainless steel crown). As described, it could well be that SE comes with a higher risk of long-term pulpal and restorative complications. Based on the mean costs for different follow-up treatments in the current SE cohort, and assuming no further costs to occur in SW at all, we estimate that SE will come with similar mean overall costs like SW only after 7 additional pulpotomies or 21 failed restorations. It is unlikely that this high number of complications will be accrued during the lifespan of the primary molars in this study.

This study has a number of strengths and limitations. First, by design, we aimed to reduce the risks of selection or detection bias. Notably, we could not blind the operators and patients. Given that the detected complications were not easy to bias, and also as the follow-up examination was performed blinded, we remain confident that the risks of bias are limited, especially as our findings are coherent with that from other studies. Second, this study reported on the restorative performance after SE versus SW using granular, validated criteria. That way, we were able to detect moderate changes in restoration quality over time and to identify possible differences in restoration deterioration, which we had otherwise not noticed if only using clinically detectable complications (requiring re-treatment) as our outcome. Third, we assessed costs, reflecting both medical and non-medical direct costs as well as indirect, opportunity costs. The latter was especially relevant, as SW, by design, generates relevant efforts for the second step, which should be reflected in any kind of economic analysis. The disadvantageous cost-effectiveness ratio of SW versus SE in our study is in line with modeling studies from the permanent dentition [23]. Last, and as a limitation, this study’s sample size was limited and this is a 2-year report, while we plan another (final) recall after 3 years of follow-up. Our findings should be seen as preliminary and overall, will require confirmation by a larger study, ideally in a practice-based setting (where re-treatments and also costs may differ). In this case, generalizability to general dental care will be higher, while notably, especially the economic analyses will never be fully independent from the setting, perspective and horizon taken. This should be borne in mind when attempting to transfer our findings to other healthcare systems or situations.

Within these limitations and after 2 years of follow-up, we did not detect significant differences in efficacy or restorative performance of SE versus SW. Notably, SW came with higher risks and costs during initial treatment, but no complications and treatment needs during follow-up, while the opposite was true for SE. The overall cost difference between therapies was large both initially and after follow-up, and it is currently not expected that SW will be as or more cost-effective than SE for managing well-defined deep carious lesions in primary molars.
